# Reversio

**Published:** 1869-12

**Authors:** 


Article V.— *Reversio.* By J. E. O'B., M.D.

Yearly zealous crusaders swoop down upon imaginary heathen to conquer for the public the holy sepulchre of medical truth.

This is a matter of known rejoicing to the storied Scovenduyck. You say to Smythje that Mrs. S. has puerperal fever; you explain the pathology of that to him in terms suited to his comprehension, and you give an outline of physiological treatment. There now flits through the sober imagination of Smythje a notion that lecturing is incompatible with action; and, *prestissimo!* you are changed for the other man.

According to Scovenduyck, Mrs. Smythje is affected with a febrifuge. The pineal gland is said by him to be in a state of morbid conjunction with the peritoneum ducts; but Smythje is informed that after the abstraction of a few gallons of blood, the application of blisters to the ears, and a few leeches to the eyelids, recovery will be certain.

Now be it observed that you have done no worse, or but little worse, than Scovenduyck in the didactic direction; but Smythje having discharged you, is mollified by Scove., who prepares the family physic for evermore. This is a matter of sublime indifference to you, to whom money is no object; but consider the disastrous consequences that may result to the Smithii. There seems to be some unnatural perverseness about sick people, which causes them to prefer of all things to have their food and medicine placed within their reach, and as little said about it as possible. They seem to imagine that their brains are not in a condition to appreciate the sublime truths with which you favor them, and they turn wearily from your syllogisms to the ministrations of the man whom experience has taught the use of those weapons whose names alone you know — to the man who is judged by the universal criterion — success.

